# Visceral Adiposity Index in Relation to Rotterdam Phenotypes of Polycystic Ovary Syndrome

**DOI:** 10.3390/biomedicines13081997

**Published:** 2025-08-16

**Authors:** Dagmara Pluta, Alicja Staśczak, Tomasz Stokowy, Maciej Migacz, Klaudia Kochman, Michał Holecki

**Affiliations:** 1 Department of Gynecological Endocrinology, School of Medicine in Katowice, Medical University of Silesia, Medyków 14, 40-752 Katowice, Poland; 2 Department of Systems Biology and Engineering, Faculty of Automatic Control, Electronics and Computer Science, Silesian University of Technology, Akademicka 16, 44-100 Gliwice, Poland; 3 Interdisciplinary Pomeranian Centre for Digital Medicine, Medical University of Gdańsk, M. Skłodowskiej-Curie 3a, 80-210 Gdańsk, Poland; 4 Department of Internal, Autoimmune and Metabolic Diseases, Faculty of Medical Sciences in Katowice, Medical University of Silesia, Medyków 14, 40-752 Katowice, Poland; 5 Student Scientific Society at the Department of Internal, Autoimmune and Metabolic Diseases, School of Medicine, Medical University of Silesia, Medyków 14, 40-752 Katowice, Poland

**Keywords:** polycystic ovary syndrome, phenotypes, lipid disorders, visceral adipose tissue, visceral adiposity index

## Abstract

Polycystic ovary syndrome (PCOS) is a hormonal disorder with complex, multifactorial and still not fully explained etiopathogenesis. It is believed that the cause is a combination of genetic and environmental factors. **Background**: With the aim to better understand PCOS etiology, the study examines body composition and compares the occurrence of lipid disorders and visceral adipose tissue depending on the adopted Rotterdam phenotypes. **Methods:** The study included 242 patients classified into four classic Rotterdam phenotypes. Clinical data from patients were collected and carefully analyzed to determine the relationship between the occurrence of lipid disorders and the visceral adiposity index (VAI). **Results:** The results obtained after assessing the differences between the Rotterdam phenotypes were not statistically significant. Differences in the levels of coefficients included in the VAI equation in the given phenotypes were also analyzed, as follows: waist circumference (*p*-value = 0.3415), BMI (*p*-value = 0.7112), TG [mmol/L] (*p*-value = 0.5341) and HDL [mmol/L] (*p*-value = 0.2302). None of the differences were statistically significant. **Conclusions:** Although the results did not show a clear association between VAI and the individual Rotterdam PCOS phenotypes, this coefficient can be used in the assessment of cardiometabolic risk in women with PCOS regardless of the adopted classification.

## 1. Introduction

Polycystic ovary syndrome (PCOS) is a common endocrinopathy of women in reproductive age that causes serious complications. Globally, it may affect 5–15% of young women, depending on the used criteria. The syndrome was first described by the American gynecologist Irving F. Stein, SR, and Michael L. Leventhal in 1935 and to this day can still be found under the historical name of Stein–Leventhal syndrome [1].

The etiopathogenesis of PCOS, although certainly complex and multifactorial, is still not fully explained [2]. The literature is dominated by three pathogenic models of polycystic ovary syndrome: the gonadotropic, ovarian, and insulin-dependent models [3]. However, there has been a tendency to examine other potential factors, such as as the role of immunological factors, neuroendocrine disruptors, growth factors, oxidative stress, adipose tissue hormones, and the role of dysbiosis of the intestinal microflora, in addition to ethnic, racial, and genetic predispositions [1,4]. PCOS is a heterogeneous and complex disease, one for which it is currently impossible to determine a single gene that is responsible for its occurrence. There is even an open genetic map of PCOS but, due to the complexity of the genetic origins of PCOS, research on it is still ongoing [5,6].

PCOS is a hormonal and metabolic disorder that is heterogeneous in terms of the occurring symptoms. The first proposal for the relevant criteria was presented in 1990 by the National Institutes of Health (NIH) and resulted in the introduction of criteria that cover the presence of all three symptoms: hyperandrogenism and/or hyperandrogenemia, oligoovulation, and the exclusion of other known causes of the above disorders, such as nonclassical adrenal hyperplasia, Cushing’s syndrome, acromegaly, hyperprolactinemia, thyroid dysfunction, or premature ovarian failure. In 2003, the Rotterdam criteria were introduced, covering two of the three symptoms, oligoovulation and/or anovulation; clinical and/or biochemical features of hyperandrogenism; and a characteristic picture of polycystic ovulation in an ultrasound examination (USG), while excluding other known causes of the above disorders. The criterion for polycystic ovary disease is met when at least one ovary contains more than 12 follicles measuring 2–9 mm or the volume of at least one ovary is greater than 10 mL [7].

According to the guidelines of the European Society of Human Reproduction and Embryology (ESHRE), the number of antral follicles that should be considered as the borderline for meeting the criterion of polycystic ovaries should be 20 [8]. Given the multitude of proposed diagnostic criteria for PCOS, its diagnosis is still a diagnosis by exclusion, although the issue regarding the list of hormonal disorders that should be excluded remains open.

### 1.1. PCOS Phenotypes

Due to the possibility of different patterns of symptoms, the following PCOS phenotypes can be distinguished:

Phenotype A: HA + PCO + O, when there is clinical and/or biochemical hyperandrogenism (HA), polycystic morphology in the ultrasound image (PCO), and menstrual disorders and associated anovulation (O).

Phenotype B: HA + O, when there is clinical and/or biochemical hyperandrogenism (HA) and menstrual disorders and associated anovulation (O) with normal ovarian ultrasound imaging.

Phenotype C: HA + PCO, when there is clinical and/or biochemical hyperandrogenism (HA) and a sign of polycystic morphology in the ultrasound image (PCO), and there are no menstrual disorders and associated anovulation.

**Figure 1:**
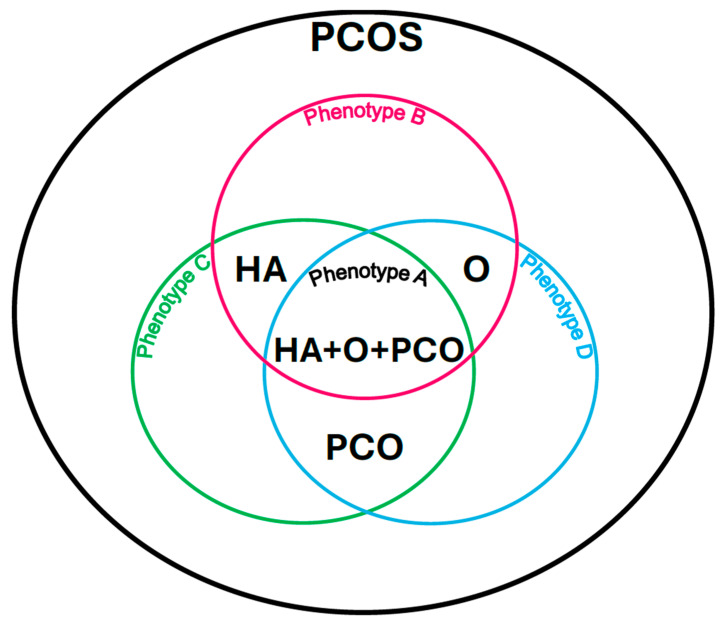
Polycystic ovary syndrome phenotypes according to the Rotterdam criteria. Own modification based on Livadas et al. [9].

Phenotype D: O + PCO, when there are menstrual disorders and associated anovulation (O) and polycystic morphology in the ultrasound image (PCO), and there are no symptoms of hyperandrogenism (HA) [9] (Figure 1).

However, considering additional parameters such as excess body weight, insulin resistance, hyperinsulinemia, we can distinguish the following phenotypes of women:

- Obese with insulin resistance.
- Obese without insulin resistance.
- Metabolically obese with normal body weight.
- Normal weight women with insulin resistance.
- Normal weight women without insulin resistance [10].

### 1.2. Metabolic Disorders Occurring in PCOS

PCOS should always be perceived as an entity combining both hormonal and metabolic disorders, especially as the latter may cause serious long-term consequences of the syndrome, such as the development of type 2 diabetes, cardiovascular complications or obesity and its consequences [1]. The most frequently mentioned symptoms accompanying PCOS include glucose intolerance disorders, type 2 diabetes, lipid disorders, hypertension, and, contributing to the pathogenesis of the syndrome, excess body weight, especially visceral obesity, insulin resistance, and hyperinsulinemia [4]. Equally common in this syndrome may be metabolic dysfunction-associated steatotic liver disease, sleep disorders, anxiety and depressive disorders, iron and other micronutrient metabolism disorders, as well as pregnancy-related complications, such as gestational diabetes or gestational hypertension [10,11,12,13].

The occurrence of metabolic disorders is frequent in this syndrome because the common link in the pathogenesis of both PCOS and metabolic syndrome is insulin resistance. Therefore, PCOS can be viewed as a gender-specific form of metabolic syndrome [14]. Metabolic syndrome can be diagnosed when three out of five criteria are met: increased waist circumference (WC), which is an expression of visceral obesity; hypertension; hyperglycemia; increased triglycerides (TG) levels; and reduced high-density lipoprotein (HDL) levels [15].

However, given the existence of multiple mechanisms linking the hepatic manifestation of metabolic syndrome with PCOS, other cholesterol fractions may also be abnormal. Excessive androgens in PCOS inhibit the expression of low-density lipoprotein (LDL) receptor RNA in both adipocytes and liver, but also very low-density lipoprotein (VLDL), potentially leading to lipid accumulation in both adipocytes and liver [11].

In addition to being the body’s largest energy store, adipose tissue is a hormonally active tissue that participates in the regulation of physiological processes through the action of adipokines such as leptin and adiponectin [16]. It has been shown that women with PCOS have more central obesity phenotypes when compared with weight- and body mass index (BMI)-matched controls. Some studies have shown that, although most women with PCOS have an android distribution of adipose tissue, they may not have an increase in visceral adipose tissue. On the other hand, gluteal and femoral body shape or gynecoid obesity, which are rare in PCOS, are independently associated with a protective lipid and glucose profile, which is associated with reduced cardiovascular and metabolic risk [17].

A new anthropometric parameter that indirectly reflects the risk of cardiometabolic complications associated with excess body weight is the visceral adiposity index (VAI). It has a similar utility to computed tomography, which is the gold standard in assessing this type of obesity [18]. VAI is a gender-dependent empirical mathematical model based on BMI, WC, TG and HDL. The described indicator is considered a marker of adipose tissue dysfunction, recommended for assessing the risk of developing circulatory system diseases, both in healthy people and those suffering from various endocrine diseases [18].

Despite the existence of many studies, it is not clear whether women with PCOS with a normal BMI have a similar pattern of metabolic disorders, including lipid disorders and adipose tissue distribution, as obese women suffering from this syndrome. It is also not clear whether women with PCOS with a normal body weight are at a similar risk of developing metabolic disorders, as is the case with obese women. Moreover, the relationship between visceral obesity depending on the phenotypes of PCOS is still not fully understood. Therefore, this work aims to investigate visceral adipose tissue depending on the phenotypes of women with PCOS, which may be helpful in better understanding the abnormal metabolic profile in this population of women and influencing its long-term consequences.

## 2. Materials and Methods

The study included 242 Caucasian women aged 18–42 years (Table 1), hospitalized in a department of gynecological endocrinology, in whom polycystic ovary syndrome was diagnosed based on the Rotterdam criteria, after a medical interview, excluding chronic diseases requiring constant treatment, gynecological examination, exclusion of pregnancy, determination of the concentration of androgens of ovarian and adrenal origin, and ultrasound examination of the reproductive organs using the Voluson 730 Expert device (GE Healthcare, Zipf, Austria). The study was based on anonymized, retrospective data collected during standard hospitalization procedures in the department of gynecological endocrinology with the approval of the bioethics committee, approval number: BNW/NWN/0052/KB/181/25. All procedures were conducted in accordance with institutional guidelines and the principles of the Helsinki Declaration. Each patient provided informed consent as part of the routine hospitalization process. No additional tests were performed beyond those routinely conducted during standard medical care. In all of the examined patients, in the morning on an empty stomach, 12 h after their last meal, a subjective and physical examination was performed, including measurements of body weight, height, WC, hip circumference and blood for the laboratory tests described below. The waist-to-hip ratio (WHR) was calculated and, following its outcome, was also defined. Body weight was assessed based on the BMI index according to the WHO criteria, which led to the diagnosis of underweight with a BMI < 18.5 kg/m^2^, normal body weight with a BMI of 18.5–24.9 kg/m^2^, overweight with a BMI of 25–29.9 kg/m^2^, first-degree obesity with a value of 30–34.9 kg/m^2^, second-degree obesity 35–39.9 kg/m^2^, and third-degree obesity (morbid) when the BMI value is ≥40 kg/m^2^ [19]. Lipid profile and serum glucose concentration were measured using colorimetric methods (AU 680 analyzer with Beckman Coulter reagents (Brea, CA, USA)). Concentrations of estradiol, follicle-stimulating hormone (FSH), lutropin (LH), total and free testosterone, 17-OH-progesterone, androstenedione, cortisol, dehydroepiandrosterone sulfate (DHEAS), sex hormone binding globulin (SHBG), and insulin were determined using chemiluminescent microparticle immunoassay (CMIA) and Abbott reagents (Architect i2000SR (Chicago, IL, USA)). Insulin resistance was evaluated indirectly using the HOMA-IR coefficient (HOMA-IR = fasting serum insulin concentration (uIU/mL) × fasting serum glucose concentration (mmol/L)/22.5). Insulin resistance was diagnosed at HOMA-IR values ≥ 2.5. The free androgen index (FAI) was calculated using the following formula: FAI = (total testosterone/SHBG) × l00%. FAI < 5% was considered normal.

VAI was defined using the following formula:

$$VAI♀=\frac{WC\;[cm]}{36.58+(1.89\times BMI)}\times \frac{TG\;[mmol/L]}{0.81}\times \frac{1.52}{HDL\;[mmol/L]}$$

where: TG—triglycerides. HDL—high-density lipoprotein.

VAI < 2.52 was considered as a result not associated with a higher risk of developing metabolic syndrome [20].

Exploratory analysis of data from patients categorized into Rotterdam PCOS phenotypes (Supplementary Materials) was conducted to determine the association with lipid disorders and VAI. R version 4.4.1 [21] was used for statistical calculations. Plotly (version 4.10.4) [22] and graphics (version 4.4.1) [21] packages were used to generate graphics. The sizes of phenotypic groups were as follows: phenotype A—141, phenotype B—31, phenotype C—40 and phenotype D—30. Due to the characteristics of the data, phenotype groups were compared with each other using the Kruskal–Wallis test [23]. Differences between specific groups were evaluated using the nonparametric Wilcoxon signed-rank test for two groups in each combination to compare the medians of their distributions. The level of significance was established at 5% (0.05).

## 3. Results

**Figure 2:**
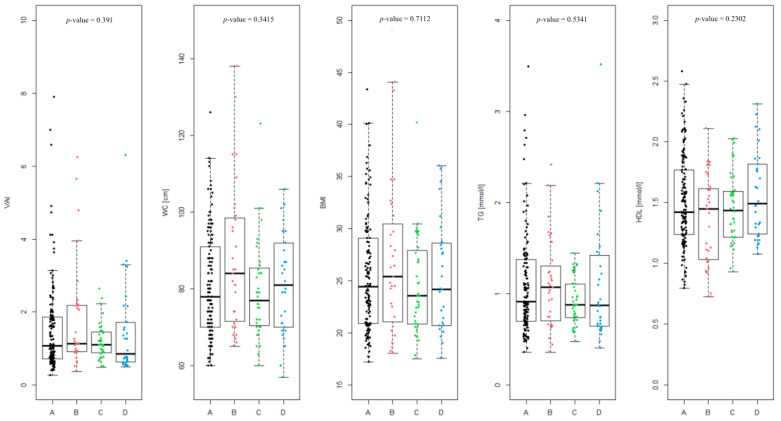
Summary of individual visceral adiposity index (VAI) values divided into Rotterdam PCOS phenotypes (A, B, C and D) and the level of coefficients in the given phenotypes included in the VAI equation (waist circumference (WC), BMI, triglycerides (TG) [mmol/L] and high-density lipoprotein (HDL) [mmol/L]).

The differences between the Rotterdam phenotypes (Figure 2) were assessed, and were found to be not statistically significant (*p*-value = 0.391). The level of differences in the coefficients included in the VAI equation in the given phenotypes was also analyzed, as follows: WC (*p*-value = 0.3415), BMI (*p*-value = 0.7112), TG [mmol/L] (*p*-value = 0.5341) and HDL [mmol/L] (*p*-value = 0.2302). None of the differences were statistically significant.

In case of phenotype A, overweight or obesity (BMI > 25) was observed in 65 of 141 patients (46.1%), WC above the norm (>80 cm) was noted in 59 patients (41.8%). A proportion of 11.4% of patients suffer from hypertriglyceridemia (TG > 1.69 mmol/L). HDL level was below the norm (<1.03 mmol/L) in 8.5% of the patients. Impaired fasting glucose (>100 mg/dL) was noted in only 4 of 139 patients (2.9%).

In phenotype B, overweight or obesity occurred in 16 of 31 patients (51.6%). WC > 80 cm was found in 19 of 31 patients (61.3%). A proportion of 9.7% of patients had elevated TG. This phenotype showed the highest proportion of patients with HDL level below the norm (25.8%). Impaired fasting glucose occurred in 4 of 30 patients (13.3%). This group has, aggregately, the highest percentage of patients exceeding given parameters standards.

Phenotype C is characterized by a lower rate of overweight or obesity, which affected 15 of 40 patients (37.5%). WC > 80 cm was also observed in 15 patients (37.5%). Elevated triglyceride level was not noted in any patients. HDL levels were within normal thresholds in almost all patients (5% of patients had HDL below the norm). Impaired fasting glucose was not observed at all in this group.

Phenotype D is characterized by overweight or obesity in 14 of 30 patients (46.7%) and WC > 80 cm in 15 patients (50%). A proportion of 13.3% of patients in this group have elevated TG levels. HDL levels were not below normal in any patient, and impaired fasting glycemia did not occur.

Phenotype B is characterized by the highest percentage of abnormal values of most metabolic parameters, which indicates a higher risk of metabolic syndrome. Phenotype C presents the most favorable results, suggesting the lowest metabolic load. Phenotype A is intermediate, while phenotype D is distinguished by a specific combination of parameters, with a high rate of central obesity and triglycerides, but without abnormalities in HDL and glucose.

**Figure 3:**
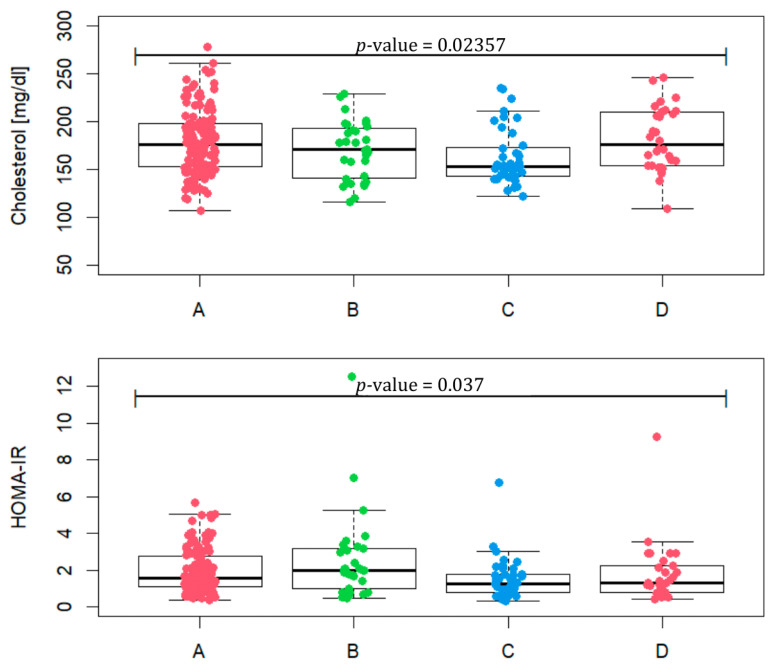
Total cholesterol level and HOMA-IR in patients representing PCOS phenotypes (A, B, C and D).

Taking into account the values, such as BMI, WC, TG, HDL and fasting glucose, included in Table 2, we checked how many patients met at least three criteria for the diagnosis of metabolic syndrome. In phenotype A, it is 12.8% of patients, in phenotype B 29%, in phenotype C 5% and in D 13.3%. Total cholesterol values between groups differ (Figure 3, *p*-value = 0.02357). A statistically significant difference occurs between phenotypes A and C (*p*-value = 0.0067) and C and D (*p*-value = 0.0056). In terms of HOMA-IR, the phenotypes assume different values (*p*-value = 0.037) for phenotypes A and C (*p*-value = 0.023) and B and C (*p*-value = 0.015). In phenotype A, four patients were excluded, and in phenotype B three were excluded, due to the lack of HOMA-IR values. The median of phenotype B reaches the value of 1.97, which may indicate the most frequent occurrence of insulin resistance in this group.

## 4. Discussion

PCOS is a hormonal and metabolic syndrome and, despite the lack of metabolic disorder markers in the diagnostic criteria of the syndrome, it should be perceived as one of them [8]. Complex etiology, heterogeneous clinical picture with reproductive, metabolic, and psychological symptoms cause women around the world to experience prolonged diagnosis and dissatisfaction with care, which translates into the occurrence of serious complications. Numerous studies show a strong correlation between the occurrence of various metabolic disorders and increased cardiovascular risk. [24]. Insulin resistance is a known, independent risk factor for cardiovascular diseases. It is directly promoted by the presence of excessive adipose tissue, which, regardless of the presence or absence of obesity, often occurs in PCOS in a visceral location. In turn, losing 5–10% of body weight improves insulin sensitivity, reduces insulin resistance, increases the number of spontaneous ovulations and reduces hyperandrogenism in this group of women [25,26]. Therefore, routine testing of BMI, WC, lipid and glucose levels, and blood pressure is of great value in preventing cardiometabolic diseases in patients with PCOS.

However, known and available anthropometric indices, such as BMI, WC or WHR, have been found to be ineffective due to their limitations regarding the proper distribution of adipose tissue. BMI does not take into account the percentage of muscle and adipose tissue and is not specific to gender and ethnicity [27]. WC shows a weak correlation in patients with PCOS without obesity. People of different heights may have the same WC, but they will not have the same risk of developing metabolic disorders [25].

However, to circumvent at least some of the known limitations, new indicators are being sought in predicting cardiometabolic events. One of these is VAI, the advantage of which is its combination of both physical parameters (BMI and WC) and metabolic parameters (TG and HDL), making it more sensitive and reliable. Commonly known BMI and WC may not distinguish between visceral and subcutaneous adipose tissue [26,28]. VAI is one of the best predictors of metabolic syndrome and can be considered an independent factor in the prevalence of this syndrome [25,29]. At the same time, it is a simple enough indicator that it can be used in everyday clinical practice and demographic studies to measure cardiometabolic risk in terms of dyslipidemia and insulin resistance.

Numerous studies have confirmed the relationship between VAI and body weight. The authors emphasize that VAI may be a good predictor of IR and hyperandrogenemia in overweight/obese women with PCOS. Such a division of patients into two groups, an overweight and/or obese group and one with normal body weight, is commonly discussed [25]. Few studies present the relationship between metabolic disorders and adipose tissue content using alternate forms of group division [30]. In the presented work, most phenotypes (A, B, D) were characterized by a high percentage of overweight and obese patients, of which the highest BMI and WC were noted in phenotype B. This may indicate a stronger relationship with metabolic dysfunction associated with excess visceral adipose tissue in this phenotype.

In the research conducted, the highest IR and fasting glucose values were presented by patients assigned to the Rotterdam phenotype B. The largest number of patients meeting at least three out of five criteria for the diagnosis of metabolic syndrome belonged to phenotype B. This phenotype was also characterized by the highest percentage of abnormal HDL results. Untypically, hypertriglyceridemia was the most common in phenotype D. Phenotype B was therefore the most burdened in terms of some abnormal metabolic parameters, excess visceral adipose tissue, and carbohydrate disorders, which indicates a significantly higher risk of cardiovascular complications and additionally emphasizes the metabolic burden of these patients. Analysis of the presented results indicates that patients with PCOS, regardless of the Rotterdam phenotype, have an increased risk of various lipid and metabolic disorders associated with the presence of excessive visceral adipose tissue.

In this study, no clear, statistically significant association was found between VAI and individual Rotterdam PCOS phenotypes. The strongest association was found in phenotypes with oligomenorrhoea (A, B, D), similar to the study by Amato et al. in which, the group of studied women with PCOS, all phenotypes with oligomenorrhea, showed a higher VAI score than the control group [31]. Similarly, Shreenidhi et al. showed that women with the full-symptom phenotype have the highest risk of developing MetS based on the studied coefficient [32]. However, in contrast to the study by Bir et al., this study did not show an association between the VAI score and phenotypes with hyperandrogenism. The aforementioned authors, comparing women with PCOS with hyperandrogenism with women without androgen excess, noted that certain obesity indicators and their optimal cut-off values differ [25]. In the results of this study, VAI was the highest in phenotypes with hyperandrogenism A and B, but at the same time was elevated in phenotype D (without hyperandrogenism) and the lowest in phenotype C (with hyperandrogenism, without oligomenorrhoea).

The study showed the smallest differences in WC, BMI, TG and HDL concentration among all phenotypes, which additionally emphasizes the lack of relationship between VAI components and the adopted division into four classic PCOS phenotypes (Figure 2). It has been demonstrated that women with PCOS, irrespective of their Rotterdam phenotype or body weight, exhibit a similar pattern of metabolic disturbances. Consequently, the assessment of body composition using the most objective and accurate methods may contribute to a faster and more precise identification of abnormal metabolic profiles, thereby facilitating appropriate therapeutic decisions and potentially mitigating long-term health consequences.

### Limitations

The limitations of this study include the lack of a control group and the small number of patients with a normoandrogenic phenotype compared with hyperandrogenic phenotypes, resulting in underrepresentation of the above-mentioned group. An additional important limitation is the young age of the patients, who may not have yet developed metabolic disorders, including lipid disorders. A theoretical limitation could be that some diseases, such as diabetes or hypertension, require appropriate treatment. However, chronic metabolic diseases were an exclusion criterion for the study.

## 5. Conclusions

Clinical practice in the assessment and treatment of PCOS remains inconsistent, with persistent key gaps between evidence and practice. Therefore, searching for a common, perhaps metabolic denominator of PCOS will allow us to look at this syndrome from the perspective of another division, not necessarily the one used so far for the Rotterdam PCOS phenotypes. The presented results did not show a clear association between VAI, lipid disorders and individual Rotterdam PCOS phenotypes. They only showed a relationship when taking into account the division into phenotypes with oligomenorrhoea. They did not show an association with the division into phenotypes with and without hyperandrogenism. It may therefore be necessary to create a new division of PCOS that takes into account metabolic parameters such as VAI. However, this coefficient can be used as a screening or complementary tool in the assessment of cardiometabolic risk in women with PCOS, regardless of the adopted division into classic PCOS phenotypes, and serve to implement preventive or therapeutic interventions.

## Figures and Tables

**Table 1:** Baseline characteristics of study participants according to PCOS phenotype (A–D). Statistical analysis was performed using the Kruskal–Wallis test. Bold value indicate statistical significance.

Parameter	Phenotype A (Mean ± SD)	Phenotype B (Mean ± SD)	Phenotype C (Mean ± SD)	Phenotype D (Mean ± SD)	*p*-Value
Age [years]	25.35 (SD = 4.96)	26.32 (SD = 4.48)	24.45 (SD = 4.74)	25.97 (SD = 5.58)	0.3349
Weight [kg]	68.72 (SD = 14.02)	74.79 (SD = 21.67)	67.02 (SD = 13.22)	71.63 (SD = 16.63)	0.5266
Height [cm]	164.97 (SD = 6.2)	165.64 (SD = 5.93)	164.97 (SD = 6.05)	166.56 (SD = 6.06)	0.6866
BMI [kg/m^2^]	25.29 (SD = 5.11)	27.29 (SD = 8.03)	24.62 (SD = 4.65)	25.77 (SD = 5.64)	0.7112
Waist circumference [cm]	80.510(SD = 12.86)	87.79 (SD = 19.58)	79.45 (SD = 12.61)	82.77 (SD = 13.4)	0.3415
Hip circumference [cm]	100.15 (SD = 9.69)	105.18 (SD = 16.38)	99.95 (SD = 10.34)	101.42 (SD = 11.92)	0.7982
Waist–hip ratio	0.8 (SD = 0.08)	0.83 (SD = 0.08)	0.79 (SD = 0.07)	0.81 (SD = 0.07)	0.1694
PBF [%]	32.63 (SD = 8.84)	35.15 (SD = 10.41)	32.62 (SD = 9.08)	32.67 (SD = 8.57)	0.4795
Insulin 0’ [µIU/mL]	8.9 (SD = 5.1)	10.82 (SD = 8.48)	6.45 (SD = 3.09)	8.98 (SD = 7.89)	0.8497
HOMA-IR	1.9 (SD = 1.13)	2.54 (SD = 2.46)	1.38 (SD = 0.73)	1.94 (SD = 1.73)	**0.037**
Glucose 0’ [mg/dL]	85.57 (SD = 5.99)	91.53 (SD = 13.43)	85.01 (SD = 6.57)	86.57 (SD = 6.8)	0.6449
Glucose 120’ [mg/dL]	108.45 (SD = 32.45)	113.78 (SD = 37.07)	111.31 (SD = 22.1)	105.42 (SD = 29.47)	0.7635
TSH [µIU/mL]	3.71 (SD = 20.6)	2.13 (SD = 0.89)	2.0 (SD = 0.94)	2.3 (SD = 1.12)	0.1669

**Table 2:** Summary of individual metabolic parameters (BMI, WC, TG, HDL, and fasting glucose) and the percentage of subjects exceeding established clinical thresholds across Rotterdam phenotypes [%]. Due to missing data on fasting glucose, some patients were excluded from the analysis (phenotype A: *n* = 139; phenotype B: *n* = 30; other groups unchanged). Accepted clinical thresholds: BMI < 25 kg/m^2^, WC < 80 cm, TG < 1.69 mmol/L, HDL > 1.03 mmol/L, fasting glucose < 100 mg/dL, and VAI < 2.52. Statistical comparisons were performed to determine whether the prevalence of values exceeding clinical norms differed significantly among Rotterdam phenotypes (A–D). The chi-squared test of independence was applied when all expected counts were ≥5; otherwise, Fisher’s exact test was used.

	Feature		BMI *p*-Value: 0.7285	WC *p*-Value: 0.2111	TG *p*-Value: 0.0747	HDL *p*-Value: 0.0038	Glucose 0’ *p*-Value: 0.0338	VAI *p*-Value: 0.149
Phenotype								
A			46.1%	41.8%	11.4%	8.51%	2.9%	14.2%
B			51.6%	61.3%	9.7%	25.8%	13.3%	16.1%
C			37.5%	37.5%	0%	5%	0%	2.5%
D			46.7%	50%	13.3%	0%	0%	13.3%

## Data Availability

All data are provided within the supplementary information file.

## Supplementary Materials

The following supporting information can be downloaded at https://www.mdpi.com/article/10.3390/biomedicines13081997/s1, Supplementary Table S1. Detailed clinical, biochemical, and hormonal data of patients participating in the study on lipid disorders in relation to Rotterdam phenotype A of polycystic ovary syndrome and visceral adiposity index; Supplementary Table S2. Detailed clinical, biochemical, and hormonal data of patients participating in the study on lipid disorders in relation to Rotterdam phenotype B of polycystic ovary syndrome and visceral adiposity index; Supplementary Table S3. Detailed clinical, biochemical, and hormonal data of patients participating in the study on lipid disorders in relation to Rotterdam phenotype C of polycystic ovary syndrome and visceral adiposity index; Supplementary Table S4. Detailed clinical, biochemical, and hormonal data of patients participating in the study on lipid disorders in relation to Rotterdam phenotype D of polycystic ovary syndrome and visceral adiposity index.

## References

[1] Christ J.P., Cedars M.I. (2023). Current Guidelines for Diagnosing PCOS. Diagnostics.

[2] Siddiqui S., Mateen S., Ahmad R., Moin S. (2022). A brief insight into the etiology, genetics, and immunology of polycystic ovarian syndrome (PCOS). J. Assist. Reprod. Genet..

[3] Radomski D., Orzechowska A., Barcz E. (2007). Present conceptions of etiopathogenesis of polycystic ovary syndrome. Ginekol. Pol..

[4] Singh S., Pal N., Shubham S., Sarma D.K., Verma V., Marotta F., Kumar M. (2023). Polycystic Ovary Syndrome: Etiology, Current Management, and Future Therapeutics. J. Clin. Med..

[5] Dapas M., Dunaif A. (2022). Deconstructing a Syndrome: Genomic Insights into PCOS Causal Mechanisms and Classification. Endocr. Rev..

[6] Kumar R., Minerva S., Shah R., Bhat A., Verma S., Chander G., Bhat G.R., Thapa N., Bhat A., Wakhloo A. (2022). Role of genetic, environmental, and hormonal factors in the progression of PCOS: A review. J. Reprod. Healthc. Med..

[7] The Rotterdam ESHRE/ASRM-Sponsored PCOS Consensus Workshop Group (2004). Revised 2003 consensus on diagnostic criteria and long-term health risks related to polycystic ovary syndrome (PCOS). Hum. Reprod..

[8] Teede H.J., Tay C.T., Laven J.J.E., Dokras A., Moran L.J., Piltonen T.T., Costello M.F., Boivin J., Redman L.M., Boyle J.A. (2023). Recommendations from the 2023 International Evidence-based Guideline for the Assessment and Management of Polycystic Ovary Syndrome. J. Clin. Endocrinol. Metab..

[9] Livadas S., Diamanti-Kandarakis E., Macut D., Pfeifer M., Yildiz B.O., Diamanti-Kandarakis E. (2013). Polycystic Ovary Syndrome: Definitions, Phenotypes and Diagnostic Approach. Frontiers of Hormone Research.

[10] Satyaraddi A., Cherian K., Kapoor N., Kunjummen A., Kamath M., Thomas N., Paul T. (2019). Body composition, metabolic characteristics, and insulin resistance in obese and nonobese women with polycystic ovary syndrome. J. Hum. Reprod. Sci..

[11] Jihad A., Sarhat E. (2023). Altered Levels of Anti-Mullerian Hormone and Hepcidin as Potential Biomarkers for Polycystic Ovary Syndrome. Georgian Med. News.

[12] Pokorska-Niewiada K., Brodowska A., Szczuko M. (2021). The Content of Minerals in the PCOS Group and the Correlation with the Parameters of Metabolism. Nutrients.

[13] Rinella M.E., Lazarus J.V., Ratziu V., Francque S.M., Sanyal A.J., Kanwal F., Romero D., Abdelmalek M.F., Anstee Q.M., Arab J.P. (2023). A multisociety Delphi consensus statement on new fatty liver disease nomenclature. J. Hepatol..

[14] Melguizo-Rodríguez L., Costela-Ruiz V.J., García-Recio E., De Luna-Bertos E., Ruiz C., Illescas-Montes R. (2021). Role of Vitamin D in the Metabolic Syndrome. Nutrients.

[15] Masenga S.K., Kabwe L.S., Chakulya M., Kirabo A. (2023). Mechanisms of Oxidative Stress in Metabolic Syndrome. Int. J. Mol. Sci..

[16] Ribeiro V.B., Kogure G.S., Lopes I.P., Silva R.C., Pedroso D.C.C., Ferriani R.A., Furtado C.L.M., Reis R.M.D. (2019). Association of measures of central fat accumulation indices with body fat distribution and metabolic, hormonal, and inflammatory parameters in women with polycystic ovary syndrome. Arch. Endocrinol. Metab..

[17] Agrawal H., Aggarwal K., Jain A. (2019). Visceral adiposity index: Simple Tool for assessing cardiometabolic risk in women with polycystic ovary syndrome. Indian J. Endocrinol. Metab..

[18] Shen F., Guo C., Zhang D., Liu Y., Zhang P. (2024). Visceral adiposity index as a predictor of type 2 diabetes mellitus risk: A systematic review and dose–response meta-analysis. Nutr. Metab. Cardiovasc. Dis..

[19] Purnell J.Q., Feingold K.R., Anawalt B., Blackman M.R., Boyce A., Chrousos G., Corpas E., de Herder W.W., Dhatariya K., Dungan K., Hofland J. (2000). Definitions, Classification, and Epidemiology of Obesity. Endotext.

[20] Amato M.C., Giordano C., Pitrone M., Galluzzo A. (2011). Cut-off points of the visceral adiposity index (VAI) identifying a visceral adipose dysfunction associated with cardiometabolic risk in a Caucasian Sicilian population. Lipids Health Dis..

[21] R Core Team (2024). R: A Language and Environment for Statistical Computing.

[22] Sievert C. (2020). Interactive Web-Based Data Visualization with R, Plotly, and Shiny.

[23] Kruskal W.H., Wallis W.A. (1952). Use of Ranks in One-Criterion Variance Analysis. J. Am. Stat. Assoc..

[24] Guan C., Zahid S., Minhas A.S., Ouyang P., Vaught A., Baker V.L., Michos E.D. (2022). Polycystic ovary syndrome: A “risk-enhancing” factor for cardiovascular disease. Fertil. Steril..

[25] Bir A., Ghosh A., Chowdhury S. (2023). Visceral adiposity index and lipid accumulation product index: The promising role in assessing cardiometabolic risk in non-obese patients of PCOS. J. Educ. Health Promot..

[26] Zhang Y., Qu Z., Lu T., Shao X., Cai M., Dilimulati D., Gao X., Mao W., Hu F., Su L. (2023). Effects of a Dulaglutide plus Calorie-Restricted Diet versus a Calorie-Restricted Diet on Visceral Fat and Metabolic Profiles in Women with Polycystic Ovary Syndrome: A Randomized Controlled Trial. Nutrients.

[27] Wu Y., Li D., Vermund S.H. (2024). Advantages and Limitations of the Body Mass Index (BMI) to Assess Adult Obesity. Int. J. Environ. Res. Public Health.

[28] Torun C., Ankaralı H., Caştur L., Uzunlulu M., Erbakan A.N., Akbaş M.M., Gündüz N., Doğan M.B., Bahadır M.A., Oğuz A. (2024). Prediction of visceral adipose tissue magnitude using a new model based on simple clinical measurements. Front. Endocrinol..

[29] Li Y., Gui J., Liu H., Guo L.-L., Li J., Lei Y., Li X., Sun L., Yang L., Yuan T. (2023). Predicting metabolic syndrome by obesity- and lipid-related indices in mid-aged and elderly Chinese: A population-based cross-sectional study. Front. Endocrinol..

[30] Sharma P., Sarkar A., Kaur H., Gupta U., Kumar B. (2022). Visceral Adiposity Index as an Indicator for Menstrual Disturbance, Hormonal and Metabolic Dysfunction in Polycystic Ovarian Syndrome. Cureus.

[31] Amato M.C., Verghi M., Galluzzo A., Giordano C. (2011). The oligomenorrhoic phenotypes of polycystic ovary syndrome are characterized by a high visceral adiposity index: A likely condition of cardiometabolic risk. Hum. Reprod..

[32] Shreenidhi R.A., Mahey R., Rajput M., Cheluvaraju R., Upadhyay A.D., Sharma J.B., Kachhawa G., Bhatla N. (2024). Utility of Visceral Adiposity Index and Lipid Accumulation Products to Define Metabolically-Unhealthy Polycystic Ovary Syndrome in Asian Indian Women—A Cross Sectional Study. J. Hum. Reprod. Sci..

